# SHP2-mediated mitophagy boosted by lovastatin in neuronal cells alleviates parkinsonism in mice

**DOI:** 10.1038/s41392-021-00474-x

**Published:** 2021-01-29

**Authors:** Wen Liu, Meijing Wang, Lihong Shen, Yuyu Zhu, Hongyue Ma, Bo Liu, Liang Ouyang, Wenjie Guo, Qiang Xu, Yang Sun

**Affiliations:** 1 State Key Laboratory of Pharmaceutical Biotechnology, Department of Biotechnology and Pharmaceutical Sciences, School of Life Sciences, Nanjing University, Nanjing, China; 2 Jiangsu Key Laboratory of Efficacy and Safety Evaluation of TCM, Nanjing University of Chinese Medicine, Nanjing, China; 3 State Key Laboratory of Biotherapy and Cancer Center, West China Hospital, Sichuan University, Chengdu, China; 4 Jiangsu Key Laboratory of New Drug Research and Clinical Pharmacy, Xuzhou Medical University, 209 Tongshan Road, Xuzhou, Jiangsu China; 5 Chemistry and Biomedicine Innovation Center (ChemBIC), Nanjing University, Nanjing, China

**Keywords:** Cell biology, Neurological disorders

**Dear Editor**,

The loss of PINK1/Parkin-dependent mitochondrial clearance causes loss of dopaminergic neurons in the substantia nigra and contributes to the pathogenesis of Parkinson’s disease (PD).^1^ Several kinases were reported to regulate the ubiquitin E3 ligase activity of Parkin through phosphorylation but the involvement of protein tyrosine phosphatase (PTPase) for Parkin activity remains elusive.^2^

Although the roles of Src homology 2 domain-containing tyrosine phosphatase-2 (SHP2) in development, hematopoiesis and cancer immunology have been intensively reported,^3,4^ knowledge of regulation and function of SHP2 in neuronal diseases remains scant. We previously showed that SHP2 maintains mitochondrial homeostasis through dephosphorylating ANT1 at Tyr-191 during NLRP3 inflammasome activation in macrophages.^5^ This previous study prompted us to investigate whether SHP2 regulates mitophagy and mitochondrial quality in neurons and, if so, whether targeting SHP2 could be a novel strategy for neuronal protection in PD.

As shown in Fig. 1a, b and Supplementary Fig. 1a–b, CCCP-induced mitochondria ubiquitination, reduction of mitochondrial mass as well as TOM20 ubiquitination and degradation were attenuated by SHP2 knockdown. Mitophagic flux examined by mt-Keima also suggests that SHP2 positively regulates mitophagy (Fig. 1c, d, Supplementary Fig. 1c–f). Next, the mitochondrial translocation of Parkin and TOM20 degradation was remarkably decreased after SHP2 knockdown (Fig. 1e, Supplementary Fig. 2a–b). Parkin ubiquitination induced by CCCP treatment was also significantly inhibited by SHP2 knockdown and augmented after SHP2 overexpression (Fig. 1f, Supplementary Fig. 2c). Coimmunoprecipitation assay showed that both endogenous SHP2 (Fig. 1g) and exogenously SHP2 interacted with Parkin (Supplementary Fig. 3a). SHP2 and Parkin colocalization in the mitochondria was validated by Structured Illumination Microscopy (SIM) and immunoblot of mitochondrial and cytosolic fractions (Supplementary Fig. 3b–d). The PTP domain of SHP2 was shown to interact with Parkin (Supplementary Fig. 3e). Moreover, the interaction of SHP2 and Parkin as well as its involvement in mitophagy was also confirmed in primary neuron cells (Supplementary Fig. 4). These findings demonstrate that SHP2-Parkin interaction is required for Parkin-mediated mitophagy.

**Fig. 1:**
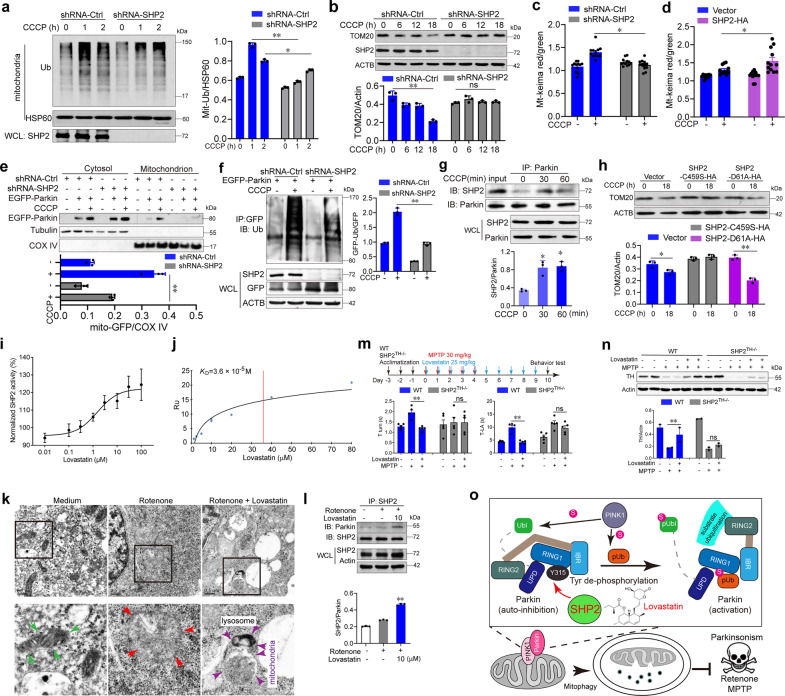
SHP2-mediated mitophagy enhanced by lovastatin in neuronal cells alleviates parkinsonism in mice. **a** Immunoblot analysis of the ubiquitination of mitochondria in shRNA-SHP2 or shRNA-Ctrl SH-SY5Y cells treated with 10 μM CCCP for indicated times. **b** Immunoblot analysis of TOM20 degradation in shRNA-SHP2 or shRNA-Ctrl SH-SY5Y cells in the presence of 10 μM CCCP for indicated times. **c**, **d** Quantification of mt-Keima (red/green) in SH-SY5Y cells with SHP2 knockdown or overexpression. The cells were treated with 10 μM CCCP for 1 h and then imaged with 458 nm (green) or 543 nm (red) light excitation. **e** Immunoblot analysis of Parkin’s mitochondrial translocation in shRNA-SHP2 or shRNA-Ctrl SH-SY5Y cells transfected with EGFP-Parkin for 24 h in the presence of 10 μM CCCP for 1 h. **f** Immunoblot analysis of Parkin ubiquitination in HeLa cells co-transfected with shRNA-SHP2 and EGFP-Parkin for 24 h followed by 10 μM CCCP treatment for 1 h. **g** Co-immunoprecipitation (Co-IP) analysis for the interaction of SHP2 with Parkin in SH-SY5Y cells treated with 10 μM CCCP for indicated times. **h** Immunoblot analysis of TOM20 expression from SH-SY5Y cells transfected with vector or SHP2-C459S or SHP2-D61A as indicated in the presence of 10 μM CCCP for 18 h. **i** The purified SHP2 protein was incubated with different doses of lovastatin and then the SHP2 enzyme activity was examined. **j** Interaction between lovastatin and SHP2 was determined via SPR analysis. **k** SH-SY5Y cells were pretreated with 10 μM lovastatin for 3 h followed by the addition of 30 μM rotenone for 6 h. Cells were collected for transmission electron microscopy assay. Green arrow, normal mitochondria; red arrow, swollen mitochondria; purple arrow, the damaged mitochondria localized near a lysosome in the autophagolysosome. **l** Co-IP analysis of Parkin and SHP2 in SH-SY5Y cells pretreated with 10 μM lovastatin for 3 h followed by the addition of 30 μM rotenone for 6 h. **m** Overview of the experimental design and behavioral tests for WT and SHP2^TH−/−^ mice treated with MPTP and lovastatin. **n** The expressions of TH in the striatum of each group were measured using Western blot analysis. **o** The graphic illustration of the mechanism of lovastatin-driven SHP2-mediated dephosphorylation of Parkin in promoting mitophagy in neuronal cells and alleviating parkinsonism in mice. Upon the initiation of mitochondrial damage in SH-SY5Y neuronal cells, SHP2 translocates to mitochondria, where it directly interacts with Parkin and promotes its E3 ligase activity via decreasing Parkin phosphorylation, increasing mitophagy as well as neuronal cell survival. Lovastatin promotes SHP2/Parkin-mediated mitophagy and exerts neuroprotective effects in MPTP-challenged mice. Data are representative of three independent experiments (mean ± SEM). *P < 0.05, **P < 0.01 vs. indicated

Since the major activity of SHP2 relied on its PTPase activity, a PTPase gain-of-function SHP2 mutant (SHP2-D61A) and a loss-of-function SHP2 mutant (SHP2-C459S) were overexpressed in HeLa cells together with EGFP-Parkin. Mitochondrial translocation of LC3B was remarkably suppressed by SHP2-C459S and promoted by SHP2-D61A (Supplementary Fig. 5a). TOM20 clearance was evidently decreased by the overexpression of SHP2-D61A compared to vector control or SHP2-C459S (Fig. 1h, Supplementary Fig. 5b). These results suggest that the PTPase activity of SHP2 is indispensable for mitophagy regulation.

Since it functions as a PTPase, SHP2 might regulate Parkin activity through the Tyr dephosphorylation of Parkin. Consistent with a previous report, the Ser phosphorylation of Parkin was enhanced upon CCCP treatment. However, in contrast, the Tyr phosphorylation of Parkin was reduced (Supplementary Fig. 5c). Noticeably, SHP2 knockdown abolished these changes in Ser and Tyr phosphorylation (Supplementary Fig. 5d). By using the purified SHP2 and Parkin, we confirmed that SHP2 could directly bind and dephosphorylate tyrosine of Parkin (Supplementary Fig. 5e–h).

Next, we wondered whether SHP2 could be a potential target to boost Parkin-mediated mitophagy. A SHP2 enzyme activity screen system was employed for identifying compounds that could promote the catalytic activity of SHP2 (Supplementary Fig. 6a). A lactone ring structure-containing compound library (Chengdu, Biopurify) was screened and lovastatin was found to be able to elevate SHP2 activity both in a cell-free system and in cells (Fig. 1i, Supplementary Fig. 6b–c). A surface plasmon resonance (SPR) assay confirmed their interaction (*K*_D_ = 36 μM, Fig. 1j), which was further demonstrated by the increased thermal stabilization of SHP2 (Supplementary Fig. 6d–e).

To examine whether lovastatin could protect mitochondria in neurons, rotenone was utilized to mimic the pathological conditions of mitochondria in PD. Lovastatin dose-dependently increased cell viability in rotenone-treated SH-SY5Y cells (Supplementary Fig. 7a). ROS generation and mitochondrial membrane potential collapse were also suppressed by lovastatin (Supplementary Fig. 7b–c). Elevated mitophagy levels in the cells were also evidenced by red fluorescence from mt-Keima in cells treated with lovastatin (Supplementary Fig. 7d). Finally, as shown in Fig. 1k for the transmission electron microscopy (TEM) images, rotenone treatment led to significant mitochondria swollen and cristae disruption. In the lovastatin-treated group, the damaged mitochondria localized near a lysosome in the autophagolysosome and were surrounded by a double membrane, suggesting that damaged mitochondrion was removed via mitophagy.

To clarify the relationship between SHP2 enzyme activity and the ability of lovastatin to promote mitophagy, SHP2 localization after lovastatin treatment was examined. SHP2 as well as Parkin translocation to mitochondria was increased after lovastatin treatment (Supplementary Fig. 8a–c). Furthermore, lovastatin treatment triggered mitochondrial protein degradation as well as Parkin ubiquitination (Supplementary Fig. 9a–b). A significant interaction between SHP2 and Parkin was observed after lovastatin treatment (Fig. 1l, Supplementary Fig. 9c). However, its ability to promote mitochondrial protein clearance and the neuroprotective effect of lovastatin was reversed after SHP2 knockdown (Supplementary Fig. 9d–e).

To assess the protective effect of lovastatin on PD, a 1-methyl-4-phenyl-1,2,3,6-tetrahydropyridine (MPTP)-induced PD murine model (MPTP-PD) was employed. Lovastatin and levodopa were administered as depicted in Supplementary Fig. 10a-b, and a series of behavioral tests including pole test, hang test, and rotarod task for detecting coordination, grasp capability was performed. The results from these behavioral experiments demonstrated that lovastatin could improve MPTP-induced behavioral impairment. Neuron injury in the striatum induced by MPTP was also ameliorated by lovastatin (Supplementary Fig. 10c–e).

To further confirm the mechanism in vivo, the activation of SHP2, interaction between SHP2 and Parkin and occurrence of mitophagy were examined. The level of phosphorylated SHP2, the active form of SHP2, in the substantia nigra was significantly elevated after lovastatin treatment, leading to a strong interaction between SHP2 and Parkin (Supplementary Fig. 10f–g). Results from TEM showed damaged mitochondria with a swollen morphology and disrupted cristae in substantia nigra of MPTP-PD mice were remarkably ameliorated by lovastatin (Supplementary Fig. 10h). Autophagolysosomes were observed after lovastatin treatment, suggesting that lovastatin triggers mitophagy in MPTP-PD mice.

Finally, to confirm the importance of SHP2 in the neuroprotective effect of lovastatin in vivo, the mice with SHP2 knockout in TH expression neuron (SHP2^TH−/−^) were generated by crossing SHP2^flox/flox^ mice with TH-Cre transgenic mice. Behavioral improvement by lovastatin was diminished in SHP2^TH−/−^ mice (Fig. 1m). TH and Nissl staining also showed that decreased damage of neuron cells by lovastatin in WT MPTP-PD mice was reversed in SHP2^TH−/−^ MPTP-PD mice (Fig. 1n, Supplementary Fig. 11a–c). Taken together, these data provide evidence that lovastatin attenuates behavioral impairment and dopaminergic neuron loss via triggering SHP2/Parkin-mediated mitophagy in MPTP-PD mice (Fig. 1o).

In summary, this study identified SHP2 as a key regulator linking Parkin-dependent mitophagy. Mechanistically, SHP2-mediated dephosphorylation of Parkin is essential for Parkin activation. This newly defined mechanism was further confirmed in mice, and lovastatin, which is identified as a SHP2 activator, could be utilized to treat PD mice. Collectively, our findings unraveled a new mechanism by which SHP2 regulates mitochondrial homeostasis and clinical drug lovastatin as a therapeutic candidate for PD.

## Supplementary information

Supplementary material

## Data Availability

All relevant data are available from the authors and/or included in the manuscript.
