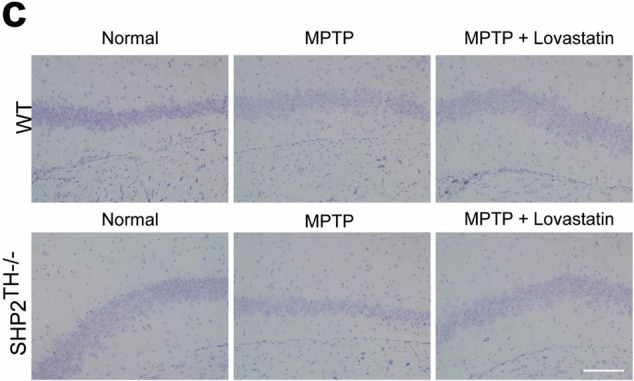# Correction: SHP2-mediated mitophagy boosted by lovastatin in neuronal cells alleviates parkinsonism in mice

**DOI:** 10.1038/s41392-024-02103-9

**Published:** 2024-12-26

**Authors:** Wen Liu, Meijing Wang, Lihong Shen, Yuyu Zhu, Hongyue Ma, Bo Liu, Liang Ouyang, Wenjie Guo, Qiang Xu, Yang Sun

**Affiliations:** 1 State Key Laboratory of Pharmaceutical Biotechnology, Department of Biotechnology and Pharmaceutical Sciences, School of Life Sciences, Nanjing University, Nanjing, China; 2 Jiangsu Key Laboratory of Efficacy and Safety Evaluation of TCM, Nanjing University of Chinese Medicine, Nanjing, China; 3 State Key Laboratory of Biotherapy and Cancer Center, West China Hospital, Sichuan University, Chengdu, China; 4 Jiangsu Key Laboratory of New Drug Research and Clinical Pharmacy, Xuzhou Medical University, 209 Tongshan Road, Xuzhou, Jiangsu China; 5 Chemistry and Biomedicine Innovation Center (ChemBIC), Nanjing University, Nanjing, China

Correction to: *Signal Transduction and Targeted Therapy* 10.1038/s41392-021-00474-x, published online 29 January 2021

In the process of collating the raw data, the authors noticed an inadvertent mistakes occurred in Supplementary Fig. 11c that need to be corrected after online publication of the article.^1^ Due to our negligence in extracting and processing a large amount of experimental data, duplicate images were inadvertently used for the MPTP group and the MPTP+ lovastatin group of mice with dopaminergic neurons deficient in SHP2 (SHP2TH-/-). The correct data are provided as follows. The key findings of the article are not affected by these corrections.

Incorrect Supplementary Fig. 11c:
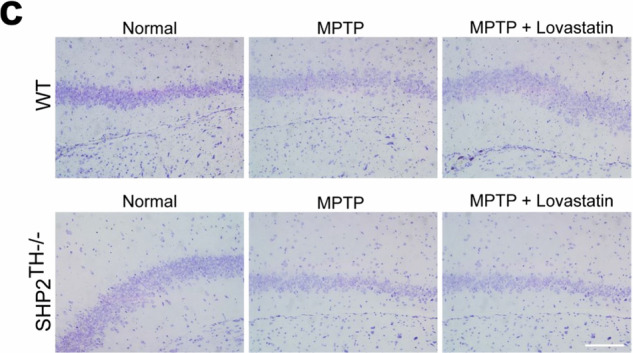


The revised Supplementary Fig. 11c is: