# Supplementary material for: SHP2-mediated mitophagy boosted by lovastatin in neuronal cells alleviates parkinsonism in mice
Source: Signal Transduct Target Ther. 2021 Jan 29;6:34. doi: 10.1038/s41392-021-00474-x (PMC7846847; doi:10.1038/s41392-021-00474-x)
Supplement: Supplementary file 1 — Supplementary material [file 41392_2021_474_MOESM1_ESM.docx]

Supplementary Materials for

SHP2-mediated mitophagy boosted by lovastatin in neuronal cells alleviates parkinsonism in mice

Wen Liu^1,#^, Meijing Wang^1,#^, Lihong Shen^1,#^, Yuyu Zhu^1^, Hongyue Ma^2^, Bo Liu^3^, Liang Ouyang^3^, Wenjie Guo^1,*^, Qiang Xu^1,*^, Yang Sun^1,4,*^

Correspondence to: Yang Sun (yangsun@nju.edu.cn) or Qiang Xu (qiangxu@nju.edu.cn) or Wenjie Guo ([guowj@nju.edu.cn](mailto:guowj@nju.edu.cn))

**This PDF file includes:**

Materials and Methods

Figures. S1 to S11

Materials and Methods

Reagents

4',6-diamidino-2-phenylindole (DAPI, D8417), Chloroquine (CQ, B1793), Carbonyl cyanide 3-chlorophenylhydrazone (CCCP, C2759) and rotenone (R8875) were purchased from Sigma-Aldrich (St. Louis, MO). Rapamycin (53123-88-9) was purchased from Aladdin (Shanghai, China). Anti-SHP2 (sc-7384), anti-TOM20 (sc-136211), anti-TOM70 (sc-39054), anti-Ub (sc-47721), anti-Parkin (sc-133167), anti-p-Ser (sc-81517) and anti-TH (sc-25269) were purchased from Santa Cruz Biotechnology (Santa Cruz, CA). Anti-Actin (M20010) and anti-Tubulin (M20005) were purchased from Abmart (Shanghai, China). Anti-COX IV (RML3033), anti-HSP60 (RML3357) and anti-GFP-tag (RML3009) were purchased from Ruiying BioTech (Suzhou, China). Anti-HA-tag (3724), anti-LC3B (2775S) and anti-p-Tyr (9411) were purchased from Cell Signaling Technology (Beverly, MA). Anti-MAP2 (17490-1-AP) were purchased from Proteintech (Wuhan, China). Mitochondria/Cytosol Fractionation Kit (ab65320) was purchased from Abcam (Cambridge, MA). Mitochondrial specific dye MitoTracker Deep Red FM (M224262), Alexa Fluor 488 goat anti-rabbit IgG (A11008), Alexa Fluor 568 goat anti-rabbit IgG (A11036) and Alexa Fluor 594 goat anti-mouse IgG (A11032) were purchased from Invitrogen (Carlsbad, CA). Nissl staining kit (C0117) were bought from Beyotime Company (Nantong, China). All other chemicals were obtained from Sigma-Aldrich.

Plasmids

Plasmid encoding mt-mKeima (mt-mKeima/pIND) was kindly provided by Dr. Atsushi Miyawaki ^1^ (Laboratory for Cell Function Dynamics, RIKEN Brain Science Institute, Japan). The mt-mKeima cDNA containing 5’-KpnI and 3’- EcoRI sites was digested and cloned into the KpnI/EcoRI sites of pcDNA3.1(+) to generate mt-mKeima/pcDNA3.1. pEGFP-Parkin-WT (45875) and pCMV-SHP2 (8381) were purchased from Addgene. PCMV-SHP2-HA, pCMV-SHP2-D61A-HA, pCMV-SHP2-C459S-HA, pCMV-SHP2-ΔPTP-HA, pCMV-SHP2-ΔSH2-HA, EGFP-SHP2 point mutation plasmid were obtained by PCR-based mutation and amplification of wild type expression vector. EGFP-Parkin-Y267A, EGFP-Parkin-Y285A, EGFP-Parkin-Y315A, EGFP-Parkin-Y318A, EGFP-Parkin-Y372A, EGFP-Parkin-Y391A were obtained by PCR-based mutation and amplification of wild type expression vector.

Cell culture

Human SH-SY5Y, HeLa and HEK293T cells were obtained from Shanghai Institute of Cell Biology (Shanghai, China). SH-SY5Y cells were cultured in RPMI 1640 and HeLa and HEK293T cells were cultured in DMEM, supplemented with 10% fetal bovine serum.

RNA interference assay

The lentiviral short hairpin RNAs (shRNAs) for SHP2 were purchased from Shanghai Obio Technology Co. Ltd. (Shanghai, China) and synthesized as following: shRNA-SHP2: 5’-ACACTGGTGATTACTATGA-3’, shRNA-Ctrl: 5’-TTCTCCGAACGTGTCACGT-3’. In our study, a dose of 50 IU lentivirus per cell was used to infect SH-SY5Y or HeLa cells. Gene silencing was performed with the following Stealth RNAi siRNAs specifically designed against HMG-CoA reductase as dexcirbed ^2^: ST-siRNA-1 (UGCUUCUACUCAUCGUGGUUGUAAA) and ST-siRNA-2 (UCUUCAUGUUAAAGGUGCUUCUGAA).

The assay for catalytic activity of SHP2

The catalytic activity of SHP2 was monitored using the surrogate substrate DiFMUP in a prompt ﬂuorescence assay format ^3^. The phosphatase reactions were performed at room temperature in 96-well black polystyrene plate, ﬂat bottom, low ﬂange, nonbinding surface (Corning, cat. no. 3575) using a ﬁnal reaction volume of 100 μl and the following assay buﬀer conditions: 60 mM HEPES, pH 7.2, 75 mM NaCl, 75 mM KCl, 1 mM EDTA, 0.05% P-20, 5 mM DTT. The tested compounds (30 μM) was incubated with SHP2 (1 nM). After 30 min incubation at 25 °C, the surrogate substrate DiFMUP (Invitrogen, cat. no. D6567, 200 μM) was added to the reaction and incubated at 25 °C for 30 min (100 μM for SHP2 1-525). The reaction was then quenched by the addition of 20 μl of a 160 μM solution of bpV (Phen) (Enzo Life Sciences cat. no. ALX-270-204). The ﬂuorescence signal was monitored using a microplate reader (TECAN, M200PRO) using excitation and emission wavelengths of 340 and 450 nm, respectively.

Cellular thermal shift assay (CETSA)

SH-SY5Y cells were incubated with or without compound for 2 h, then the cells were collected and subjected to Cellular Thermal Shift Assay (CETSA) assay ^4, 5^. Briefly, incubated cells were equally divided into 10 parts, each parts got heated for 3 min under different temperature (43, 46, 49, 52, 55, 58, 61, 64, 67, 70°C), then the heated cells were put into -80°C for 12 h, put into room temperature for 5 min, then repeated one more time. After that, cell lysates were extracted by centrifuged at 20,000 *g*, 20 min. Level of SHP2 was detected by western blot.

Molecular docking

The X-ray crystal structure of SHP2 was downloaded from Protein Databank (PDB, http://www.rcsb.org/). Subsequently, based on the active binding pocket, molecular docking of candidate compounds was performed by Accelrys Discovery Studio (version 3.5, Accelrys, San Diego, CA, USA). Candidate compounds were constructed using Discovery studio with CHARMm force field parameters. Molecular dockings were performed by CDOCKER protocol. The other parameters were fixed as default values.

Surface plasmon resonance (SPR)

SPR assay was performed using the Biacore T200 as follows. Recombinant human SHP2 protein was immobilized on a Biacore CM5 sensor chip via the primary amine groups. The compounds were flowed at a rate of 30 μl/min for 60 s to allow for association, followed by 150 s for dissociation over immobilized protein in PBS/5% DMSO running buffer (1.05×PBS, 0.5% P20 surfactant, 5% DMSO, pH 7.4). Andrographolide was tested for binding at 1.5 μM to 80 μM. Normalization of the data involved transformation of the y-axis such that the theoretical maximum amount of binding for a 1:1 interaction with the protein surface corresponded to a sensor response of 100 relative units (RU).

Microscale Thermophoresis (MST)

MST assays was performed using the Monolith NT.115 (Nano Temper). Recombinant human SHP2 protein was labeled by Monolith Protein Labeling Kit RED-NHS 2nd Generation. As for the mutant SHP2, proteins were extracted from the cells transfected with the corresponding plasmid. The fluorescence intensity was detected by Pico Red channel or Nano Blue channel, and the protein was diluted to an appropriate concentration for subsequent experiments. The diluted compounds (16 concentration gradients, 1:1 dilution) were respectively mixed with the protein and detected by capillary aspiration. Using MO. Affinity Analysis software for combined Analysis.

ROS, mitochondria membrane potential analysis

Intracellular ROS was determined using MitSox stain and mitochondrial membrane potential was measured by JC-1 stain. After treatment with andrographolide and rotenone, cells were washed with 0.01 M PBS and incubated with 20 μM DCFH-DA or 5 μg/ml JC-1 at 37°C for 30 min, then cells were analyzed using flow cytometry (BD) or measured with fluorescence spectrometry (Spectra MaxGemini, Molecular Devices Corporation, USA).

Mitochondria isolation

The cytoplasmic and mitochondrial fractions were prepared by using the Mitochondria Isolation Kit (Thermo Fisher Scientific) according to the manufacturer’s instructions. Briefly, cells were lysed by reagents A, B, and C supplied with the kit and centrifuged at 700 *g* at 4 °C for 10 min to obtain a post nuclear supernatant. The mitochondria were pelleted by centrifugation at 10,000 *g* at 4 °C for 15 min. The supernatant fraction was the cytosolic protein fraction. The various fractions were analyzed by immunoblot analysis.

mt-Keima assay

mt-mKeima/pcDNA3.1 plasmid were transfected into cells by using Lipofectamine 3000 (Thermo. Cat. L3000-015). After treatment, cells were collected and analyses by FASC (Attune NxT, Thermo). Dual-excitation ratiometric pH measurements were made using 488 nm (pH 7) and 561 (pH 4) nm lasers with 620/29 nm and 614/20 nm emission filters, respectively. Either 50,000 events were collected for each sample and subsequently gated for analysis using FlowJo (v10; Tree Star).

Immunofluorescence assay

Adherent cells on coverslips were fixed in 4% paraformaldehyde in PBS for 20 min at room temperature. Cells then permeabilized with 0.5% Triton X-100 in PBS for 20 min. After blocking with 5% BSA for 1 h, cells were incubated with primary antibodies overnight at 4 °C followed by incubation with Alexa Fluor-conjugated secondary antibody for 2 h and 1 μg/ml DAPI for 5 min. The slides were then mounted with ProLong Gold (Life Technologies) and imaged with a Leica TCS SP8 fluorescent confocal microscope.

Co-immunoprecipitation assay

Proteins from cells were incubated with 1 μg of appropriate antibody and precipitated with protein A/G-agarose beads (Santa Cruz Biotechnology, Santa Cruz, CA). The immunoprecipitated proteins were separated by SDS-PAGE and immunoblot analysis was performed with the indicated antibodies as described previously.

Western blot

The protein lysates were separated by 10% SDS-PAGE and subsequently electrotransferred onto a polyvinylidene difluoride membrane (Millipore, Bedford, MA). The membrane was blocked with 5% nonfat milk for 1 h at room temperature. The blocked membrane was incubated with the indicated primary Abs, and then with a horseradish peroxidase-conjugated secondary Ab. Protein bands were visualized using Western blotting detection system according to the manufacturer’s instructions (Cell Signaling Technology, MA).

Mice and MPTP-PD model

C57BL/6 mice (male, 10-12 weeks old, 23-25 g) were purchased from GemPharmatech Co. Ltd. (Nanjing, China). The TH expression neuron-specific knockout mice (SHP2^TH-/-^) were generated by crossing SHP2^flox/flox^ mice with TH-Cre transgenic mice. The animals were maintained with free access to pellet food and water in plastic cages at 21 ± 2 °C and kept on a 12 h light-dark cycle. All mice are in C57BL/6 background and are harbored in the specific pathogen-free facility in Nanjing University. Eight-week-old female cSHP2-KO mice and WT littermates were used. Animal welfare and experimental procedures were carried out strictly in accordance with the Guide for the Care and Use of Laboratory Animals (National Institutes of Health, USA) and the related ethical regulations of our university. All efforts were made to minimize animals' suffering and to reduce the number of animals used.

Adult male C57BL/6 mice were randomly divided into five groups (10 mice per group). In brief, control group was treated with PBS (vehicle). Mice were intraperitoneally administered with MPTP (dissolved in PBS) in a final concentration of 25 mg/kg daily for five consecutive days. One hour after MPTP injection, mice were orally administrated 12.5 or 25 mg/kg lovastatin once a day for 10 days. Levodopa was orally administrated (75 mg/kg, once a day for 10 days) as a positive drug. After the final treatment, behavioural procedures including pole test, beam hang test, rotarod task, and forced swimming tests were conducted to assess behavioural alternations as described before ^6^. All the evaluators were blinded to the groups.

Immunohistochemical analysis, TUNEL assay and Nissl staining

Immunohistochemical analysis was performed on paraffin-embedded brain tissue sections (3 μm). Briefly, the sections were deparaffinised, rehydrated, and washed in PBS, and then treated with 2% hydrogen peroxide, blocked with 3% goat serum, and incubated with anti-tyrosine hydroxylase TH (TH) (1:500) overnight at 4 °C. The slides were then processed with GTVisinTM™ anti-mouse/anti-rabbit immunohistochemical analysis KIT according to the manufacturer's instructions.

TUNEL and Nissl staining were performed to examine apoptosis and neuron damage, respectively, according to the manual.

Isolation and culture of the primary neuron cells from mice

Ventral mesencephalon was dissected from E12.5 C57BL/6 embryos in cold 0.01μM PBS on ice, and DMEM containing 0.25% trypsin was added and incubated for 10 min in a 37 °C water bath. The tissue pieces were dissociated by a 1 ml pipette tip through pipetting five to seven times. The enzymatic digestion was terminated by adding DMEM/F12 (Gibco) supplemented with 10% FBS. The cells were pelleted by centrifugation for 3 min at 500 × *g* at room temperature, seeded on 96 well plate coated with poly-D-lysine, and cultured in B27 supplemented neurobasal medium (Gibco, A3582801 and A3582901). Half of the media was replaced with fresh media every other day until the seventh day ^6^.

Protein purification

The full length of Parkin (1-465) was cloned into pcDNA3.1 with 6×His tag on C-terminal. For producing purified His-Parkin protein, His-Parkin plasmid was transiently transfected into HEK-293 cells. After 48 h, the cells were collected and lysed in lysis buffer (50 mM Hepes, pH 7.4, 150 mM NaCl, and 0.4% CHAPS) for 30 min. After sonication, the insoluble fraction was removed by centrifugation at 12,000 *g* for 15 min at 4°C. The His-Parkin protein was initially purified by incubation with nickel-nitrilotriacetic acid matrices (QIAGEN) for 45 min at room temperature. Histidine pull-down products were eluted in elution buffer after washing twice with lysis buffer. The eluted fractions were filtered through a 0.45-µm syringe filter and concentrated by the ultrafiltration device (Merck Millipore). Afterward, 5,000 µl of the final eluted fractions was injected into a Superdex 200 10/300 GLcolumn (GE Healthcare). Protein was further purified in 50 mM Hepes, pH 7.4, and 150 mM NaCl on an AKTA purifier (GE Healthcare). The protein was resolved by SDS-PAGE gels and detected by anti-Parkin antibody.

Microscale Thermophoresis (MST)

MST assays was performed using the Monolith NT.115 (Nano Temper). Recombinant human SHP2 protein was labeled by Monolith Protein Labeling Kit RED-NHS 2nd Generation. The fluorescence intensity was detected by Pico Red channel or Nano Blue channel, and the protein was diluted to an appropriate concentration for subsequent experiments. The diluted purified Parkin (16 concentration gradients, 1:1 dilution) were respectively mixed with the protein and detected by capillary aspiration. MO. Affinity Analysis software was used for combined analysis.

Statistical analysis

Data are expressed as mean ± SEM of three independent experiments and each experiment included triplicate sets. Statistically evaluated by Student’s *t*-test when only two value sets were compared and one-way analysis of variance (ANOVA) followed by Dunnett’s test when the data involved three or more groups. *P* < 0.05 was considered significant.

References

1. Katayama H, Kogure T, Mizushima N, Yoshimori T, Miyawaki A. A sensitive and quantitative technique for detecting autophagic events based on lysosomal delivery. *Chem Biol* **18:** 1042-1052(2011).

2. Martin-Navarro CM*, et al.* Inhibition of 3-hydroxy-3-methylglutaryl-coenzyme A reductase and application of statins as a novel effective therapeutic approach against Acanthamoeba infections. *Antimicrob Agents Chemother* **57:** 375-381(2013).

3. Chen YN*, et al.* Allosteric inhibition of SHP2 phosphatase inhibits cancers driven by receptor tyrosine kinases. *Nature* **535:** 148-152(2016).

4. Jafari R*, et al.* The cellular thermal shift assay for evaluating drug target interactions in cells. *Nat Protoc* **9:** 2100-2122(2014).

5. Martinez Molina D*, et al.* Monitoring drug target engagement in cells and tissues using the cellular thermal shift assay. *Science* **341:** 84-87(2013).

6. Geng J*, et al.* Andrographolide alleviates Parkinsonism in MPTP-PD mice via targeting mitochondrial fission mediated by dynamin-related protein 1. *Br J Pharmacol* **176:** 4574-4591(2019).


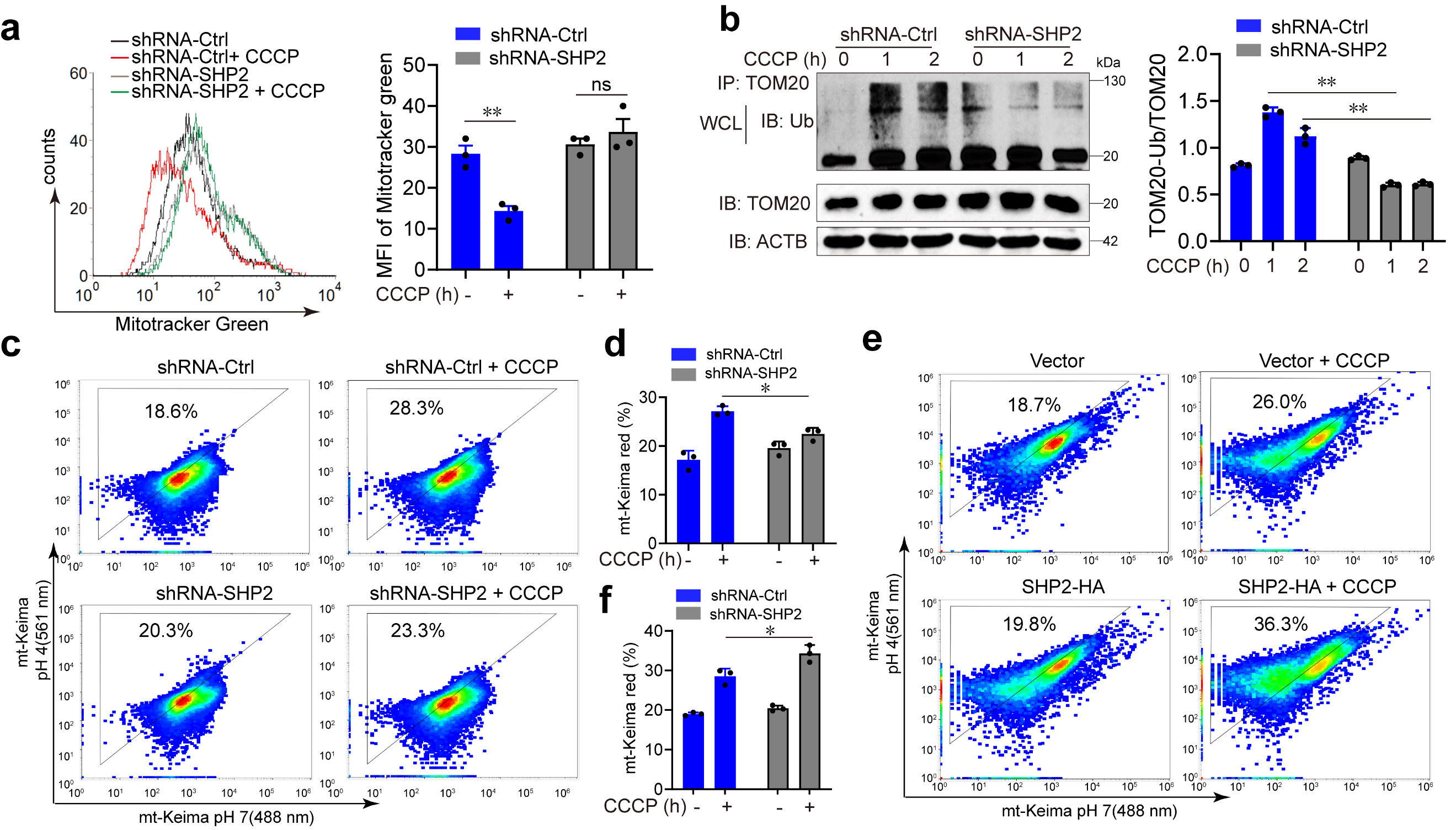


Figure. S1.

**SHP2 is required for mitophagy.** (**a**) Flow cytometric analysis of mitochondrial mass in shRNA-SHP2 or shRNA-Ctrl SH-SY5Y cells treated with 10 μM CCCP or 10 μM rotenone for 24 h by MitoTracker Green staining. (**b**) Immunoblot analysis of the ubiquitination of mitochondria in shRNA-SHP2 or shRNA-Ctrl SH-SY5Y cells treated with 10 μM CCCP for indicated times. (**c-e**) Flow cytometry analysis of mitophagy in SH-SY5Y cells with SHP2 knockdown or overexpression (transfected with mt-Keima for 48 h) after being treated with 10 μM CCCP for 1 h. Data are representative of three independent experiments (mean ± SEM). ***P*<0.01 vs indicated.


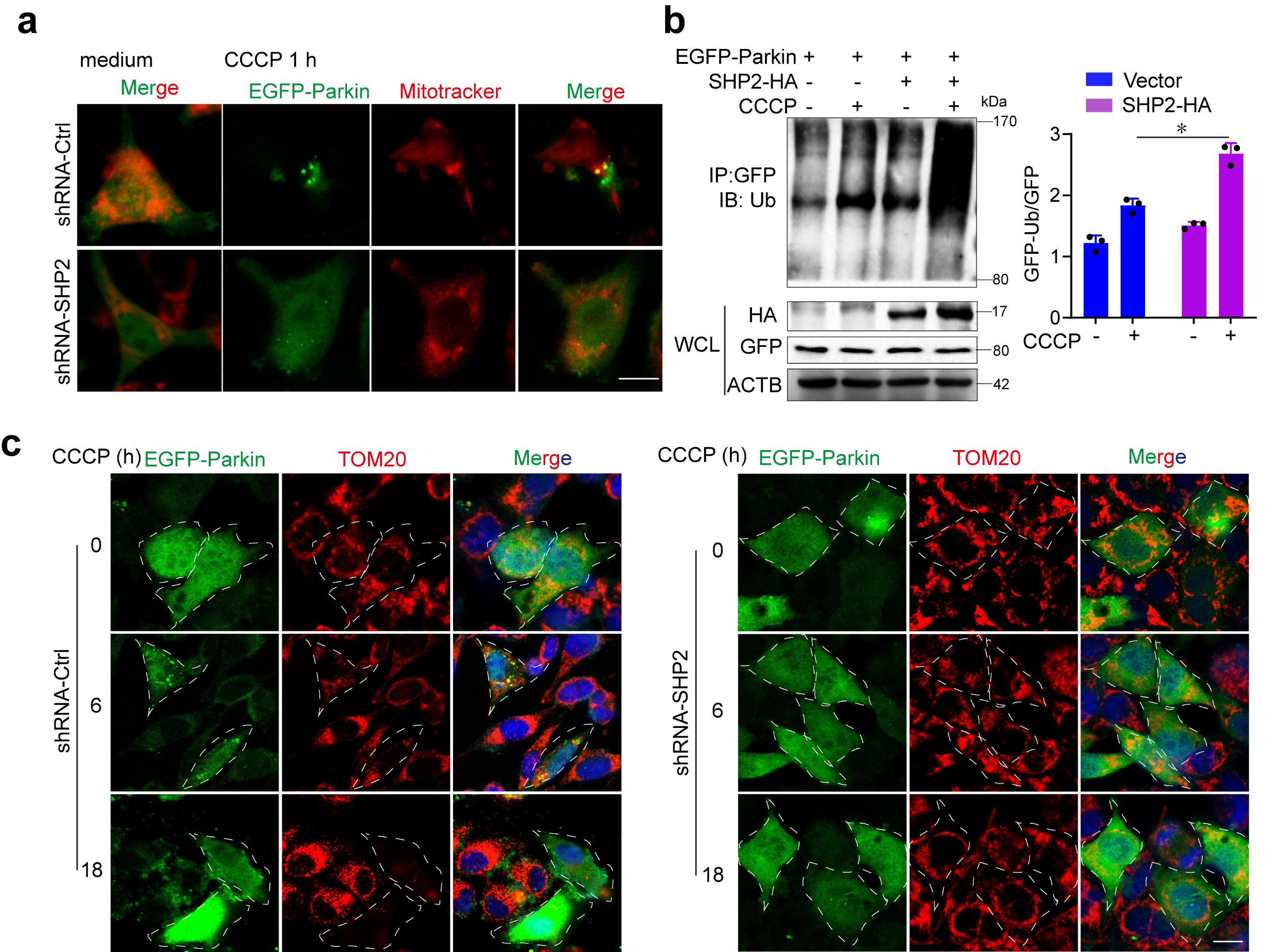


Figure. S2.

**SHP2 enhances Parkin-mediated mitochondrial clearance.** (**a**) Immunofluorescence analysis of Tom20 expression (red) and Parkin clusters (green) in shRNA-SHP2 or shRNA-Ctrl SH-SY5Y cells transfected with EGFP-Parkin for 24 h in the presence of 10 μM CCCP for indicated times. (**b**) Immunofluorescence analysis of Tom20 expression (red) and Parkin clusters (green) in shRNA-SHP2 or shRNA-Ctrl SH-SY5Y cells transfected with EGFP-Parkin for 24 h in the presence of 10 μM CCCP for indicated times. Scale bar represents 10 μm in **a** and **b**. (**c**) Co-IP analysis of Parkin ubiquitination in HeLa cells co-transfected with SHP2-HA and EGFP-Parkin for 24 h followed by 10 μM CCCP treatment for 1 h. Data are representative of three independent experiments (mean ± SEM). **P*<0.05 vs indicated.


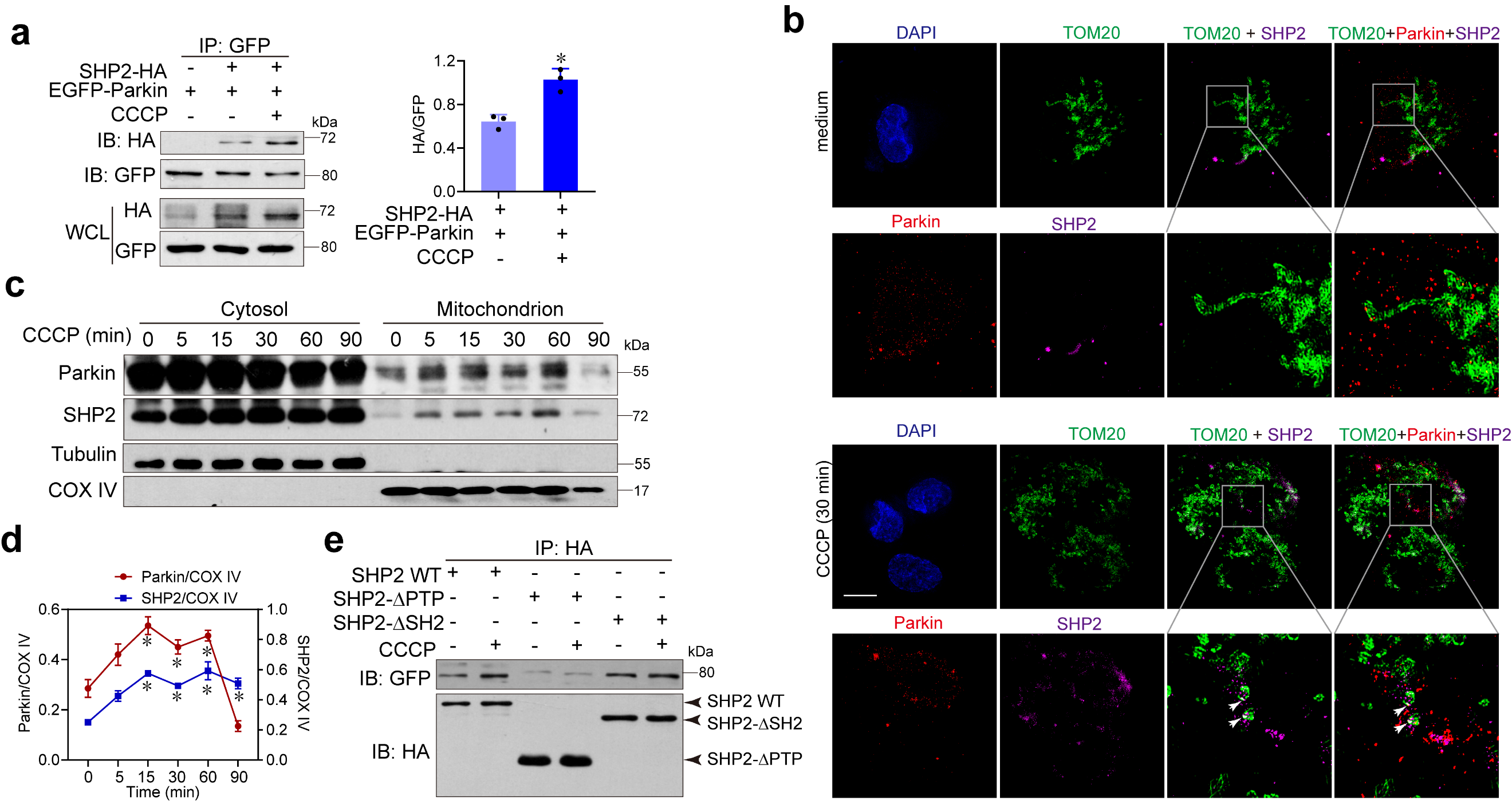


Figure. S3.

**SHP2 interacts with Parkin during mitophagy.** (**a**) Co-IP analysis for interaction of SHP2 with Parkin in 293T cells co-transfected with SHP2-HA and EGFP-Parkin in the presence of 10 μM CCCP for 30 min. (**b**) Immunofluorescence analysis of co-localization of SHP2, Parkin and mitochondrion from SH-SY5Y cells treated with 10 μM CCCP for 30 min by Structured Illumination Microscopy (SIM, Deltavision, OMX, GE). Scale bar represents 5 μm. (**c, d**) Immunoblot analysis of SHP2 and Parkin in mitochondrial and cytosolic components from SH-SY5Y cells treated with 10 μM CCCP for indicated times. (**e**) Co-IP analysis in 293T cells co-transfected with EGFP-Parkin and SHP2-WT-HA or SHP2-△PTP-HA or SHP2-△SHP2-HA plasmid respectively in the presence of 10 μM CCCP for 30 min. Data are representative of three independent experiments (mean ± SEM). * *P* <0.05. * *P* <0.01 vs indicated.


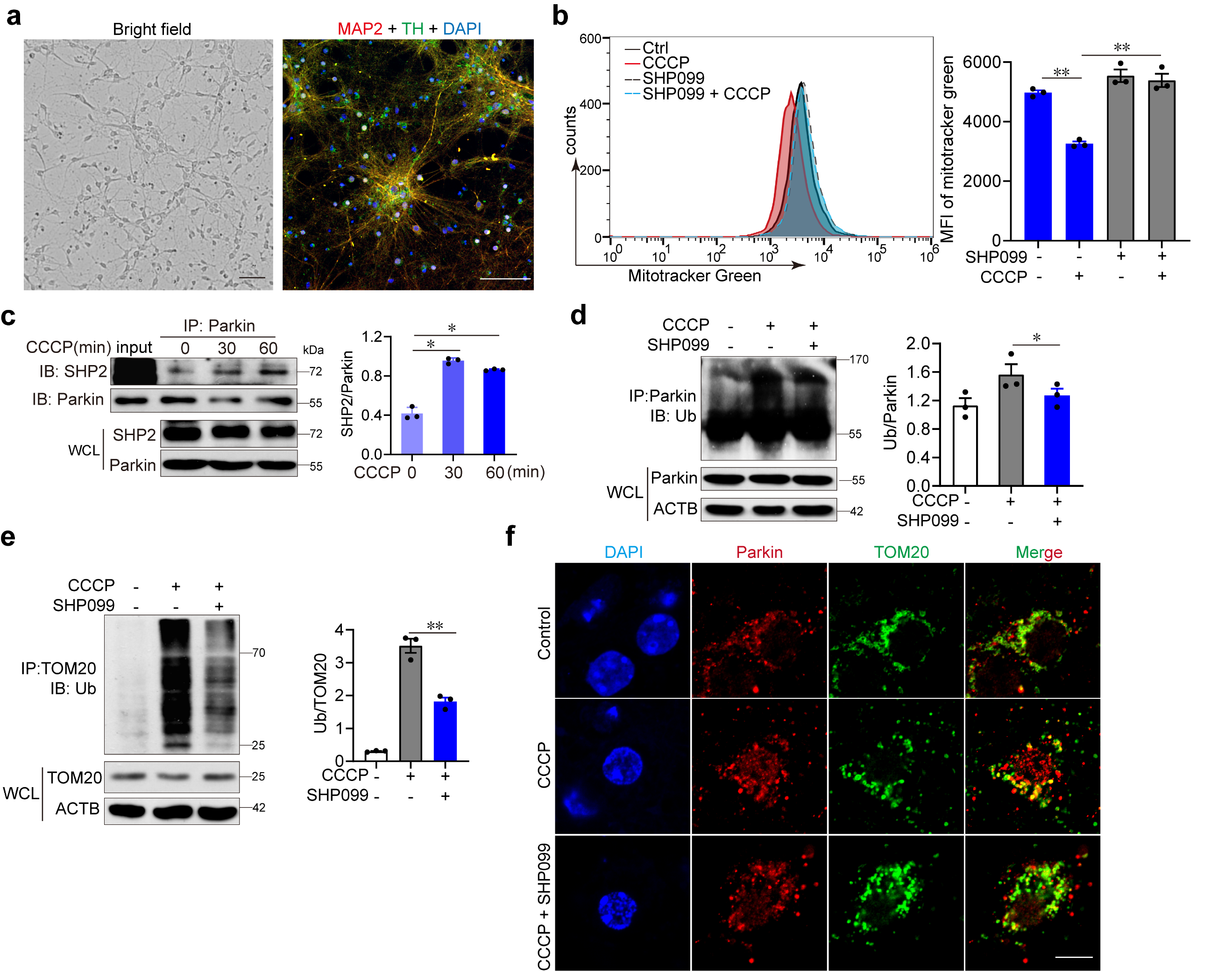


Figure. S4.

**SHP2 interacts with Parkin and affects mitophagy in primary neuron cells.** (**a**) Identification of primary neuron cells isolated from mice. Scale bar: 50 μm. (**b**) Flow cytometric analysis of mitochondrial mass in primary neuron cells treated with 10 μM SHP099 (a SHP2 specific inhibitor) or 10 μM CCCP for 24 h by MitoTracker Green staining. (**c**) Co-IP analysis for interaction of SHP2 with Parkin in primary neuron cells in the presence of 10 μM CCCP for 30 and 60 min. (**d, e**) Co-IP analysis of Parkin as well as TOM20 ubiquitination in primary neuron cells followed 10 μM SHP099 or 10 μM CCCP for 1 h. (**f**) Immunofluorescence analysis of co-localization of Parkin and mitochondrion in primary neuron cells treated with 10 μM SHP099 or 10 μM CCCP for 30 min. Scale bar represents 10 μm. Data are representative of three independent experiments (mean ± SEM). * *P* <0.05. * *P* <0.01 vs indicated.


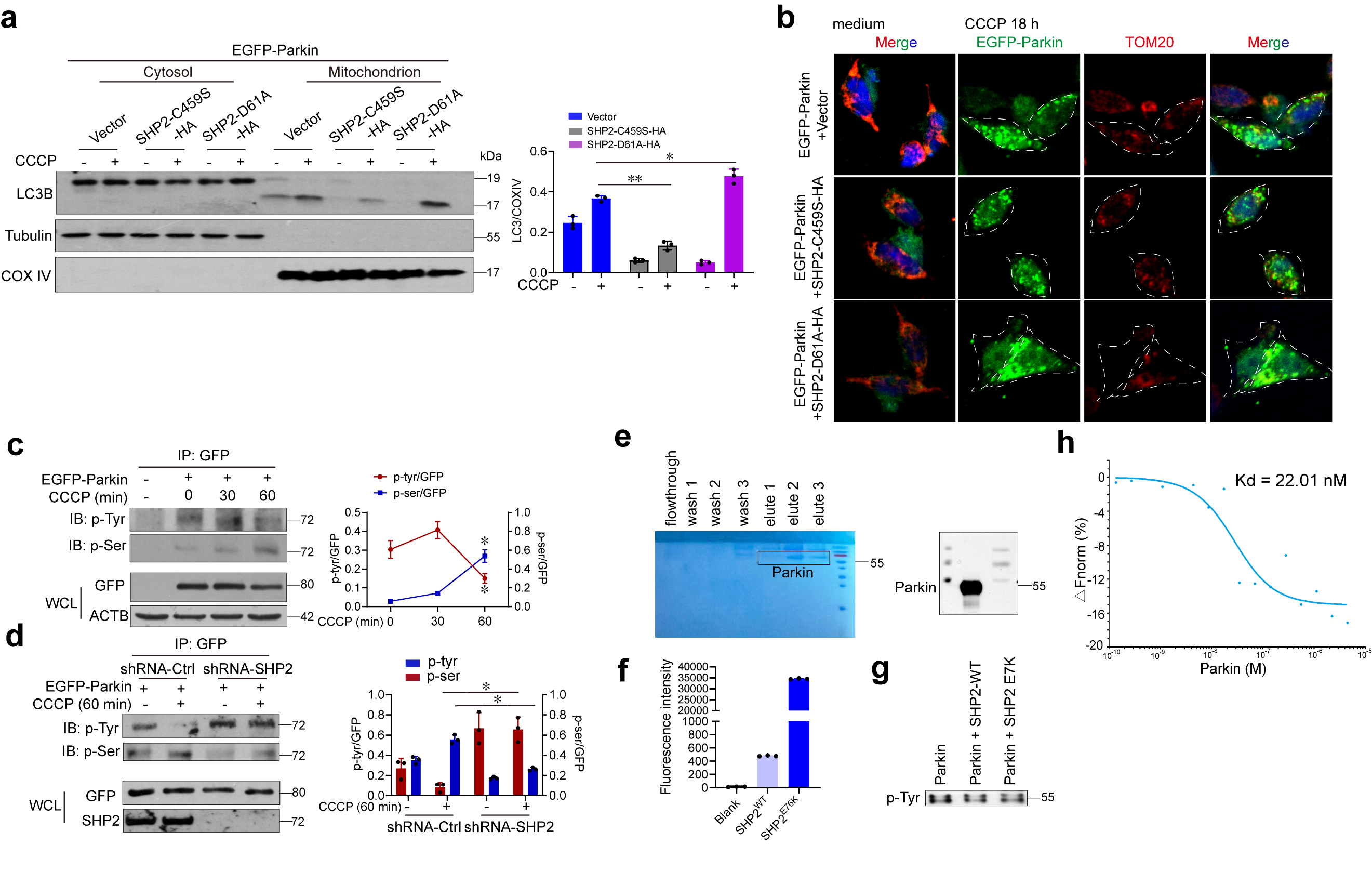


Figure. S5.

**Phosphatase activity of SHP2 is required for mitophagy regulation.** (**a**) Immunoblot analysis of LC3B in mitochondrial and cytosolic components from SH-SY5Y cells co-transfected with EGFP-Parkin and SHP2, SHP2-C459S or SHP2-D61A as indicated in the presence of 10 μM CCCP for 30 min. (**b**) Immunofluorescence analysis of TOM20 expression (red) and Parkin clusters (green) from SH-SY5Y cells co-transfected with EGFP-Parkin and SHP2, SHP2-C459S or SHP2-D61A in the presence of 10 μM CCCP for 18 h. Scale bar represents 10 μm. (**c**, **d**) Co-IP analysis of tyrosine and serine phosphorylation of Parkin in HeLa cells co-transfected with EGFP-Parkin alone (**c**) or EGFP-Parkin and shRNA-SHP2 (**d**) for 24 h followed by 10 μM CCCP treatment for 1 h. (**e**) Expression, purification and Identification of Parkin. (**f**) Enzyme activity of *E. coli*-expressed SHP2-WT and SHP-E76K were examined. (**g**) Purified Parkin were incubated with SHP2-WT or SHP2-E76K for 30 mins at 4 ℃ and then total p-Tyr of Parkin was determined by Western blot. (**h**) The interaction between SHP2 and Parkin were examined by MST. Data are representative of three independent experiments (mean ± SEM). **P*<0.05, ***P*<0.01 vs indicated. ns: not significant.


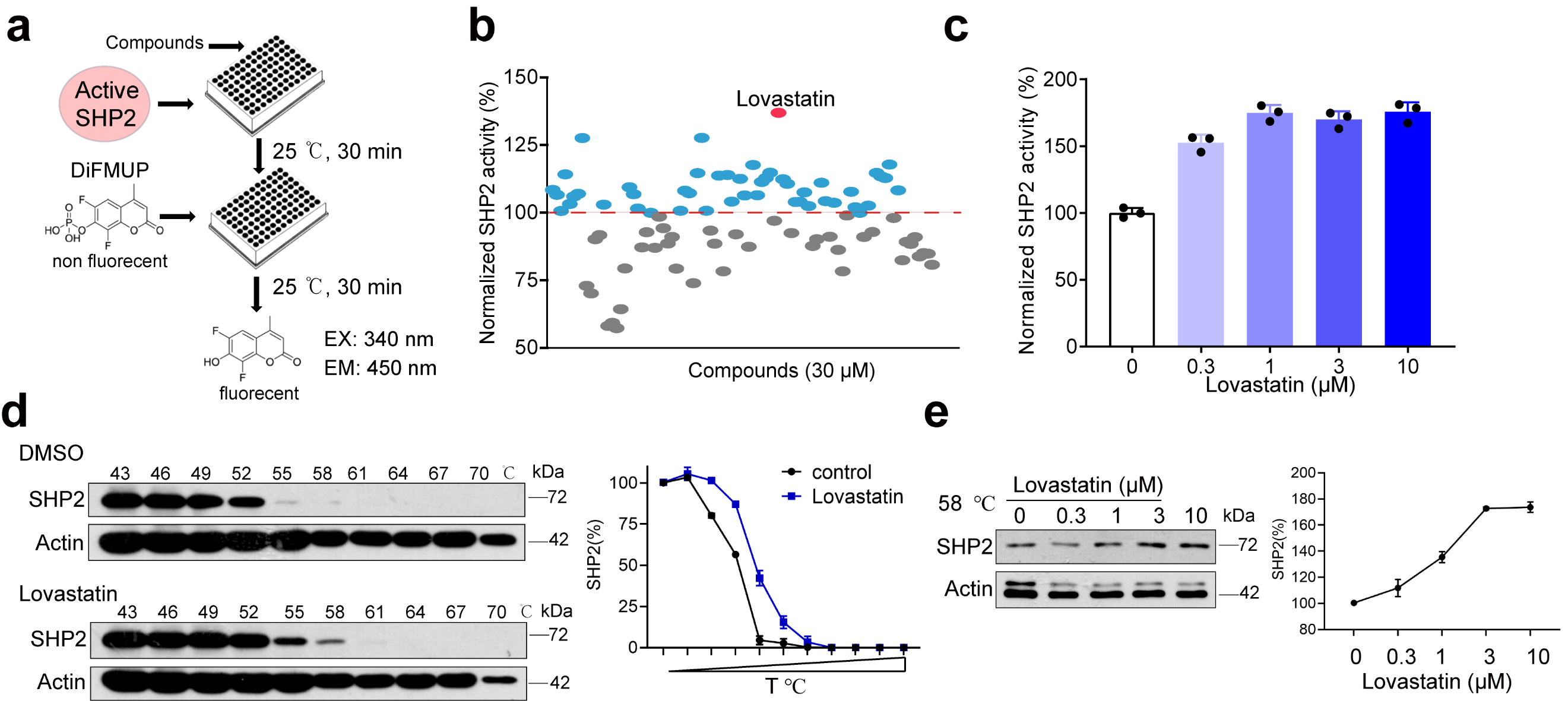


Figure. S6.

**Lovastatin promotes SHP2 PTPase activity via direct interaction.** (**a**) The process for screening of SHP2 activators. The catalytic activity of SHP2 was monitored using the surrogate substrate DiFMUP in a prompt ﬂuorescence assay format as described in the method part. **(c)** SH-SY5Y cells were incubated with or without lovastatin (10 μM) for 2 h, then the cells were collected and subjected to SHP2 enzyme activity assay. (**d**) SH-SY5Y cells were incubated with or without lovastatin (10 μM) for 2 h, then the cells were collected and subjected to SHP2 assay. (**e**) SH-SY5Y cells were incubated with or without indicated dose of lovastatin for 2 h, then the cells were collected and subjected to CETSA assay at 58 ℃. Data are representative of three independent experiments (mean ± SEM).


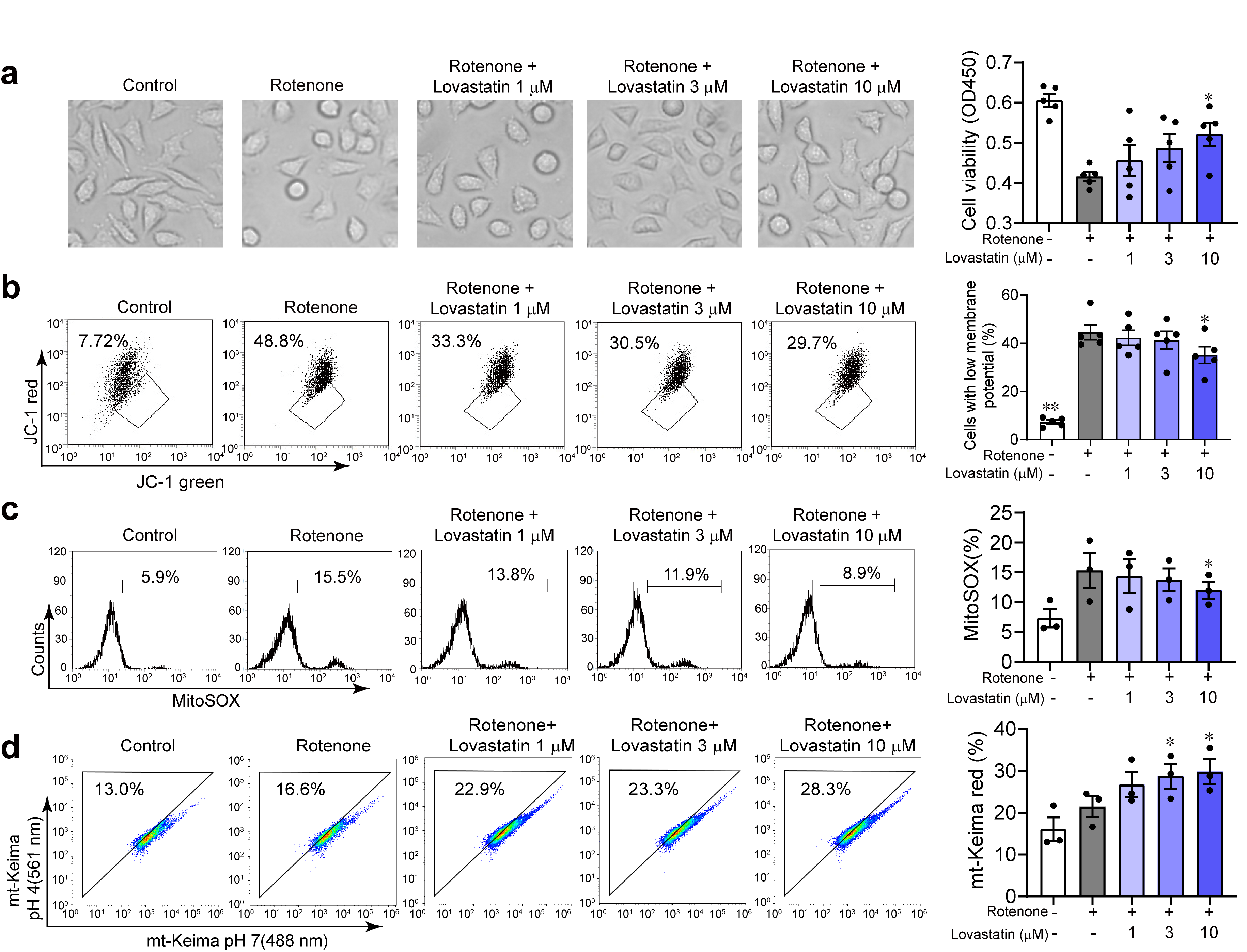


Figure. S7.

Lovastatin protects neuron cells from mitochondria damage. (a) SH-SY5Y cells were pretreated with 1, 3, 10 μM lovastatin for 3 h followed by the addition of 30 μM rotenone for 6 h, the morphological changes were pictured and cell viabilities were measured by CCK8 assay. (b, c) Flow cytometry analysis of mitochondrial ROS by MitoSOX staining or mitochondrial membrane potential by JC-1 staining in SH-SY5Y cells after being pretreated with with 1, 3, 10 μM lovastatin for 3 h followed by the addition of 30 μM rotenone for 6 h. Data are representative of three independent experiments (mean ± SEM). **P*<0.05 vs. as rotenone-treated group. (d) Flow cytometry analysis of mitophagy in SH-SY5Y cells (transfected with mt-Keima for 48 h) after being pretreated with 1, 3, 10 μM lovastatin for 3 h followed by the addition of 30 μM rotenone for 6 h.


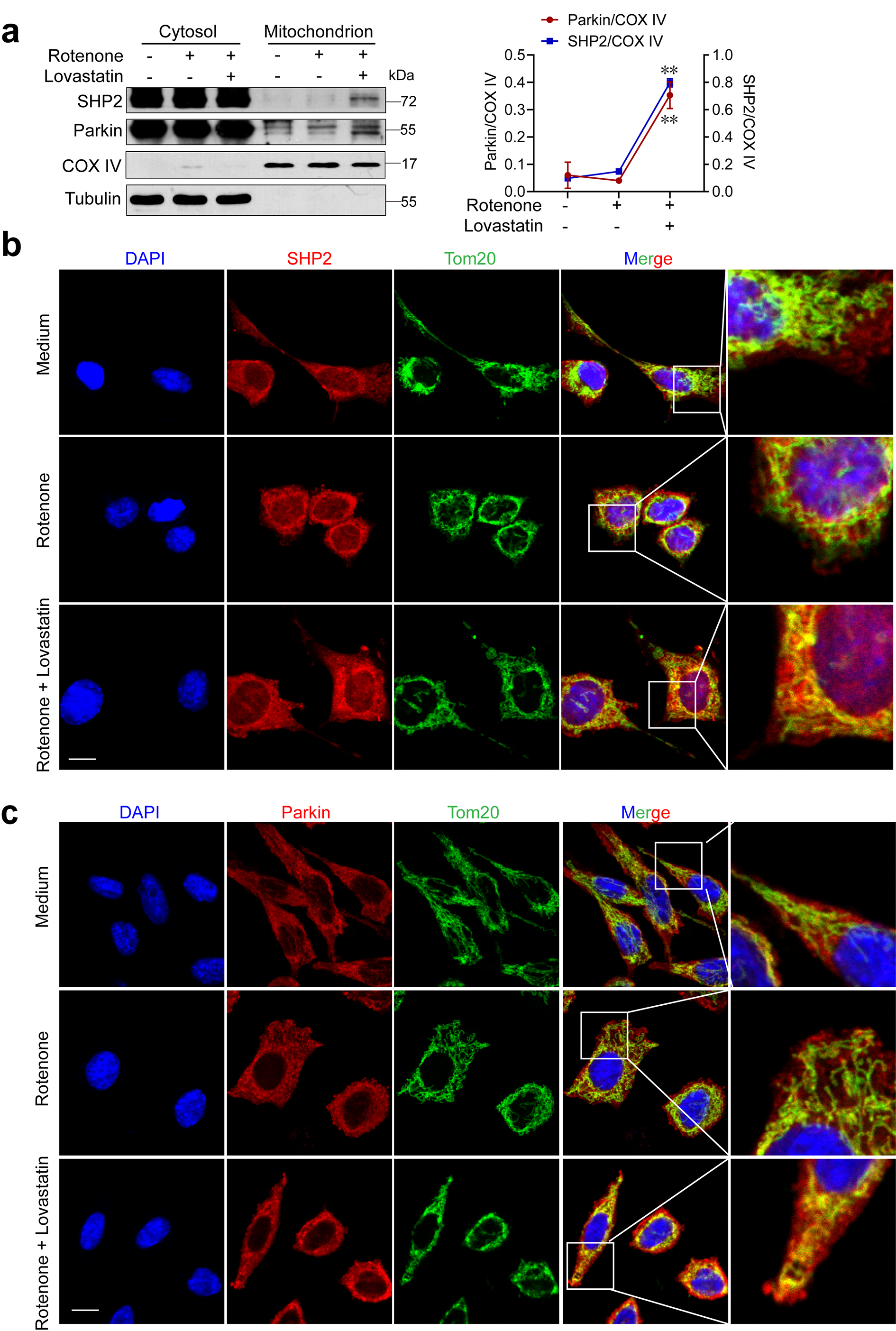


Figure. S8.

Lovastatin promotes SHP2 mitochondrial localization. (a) Immunoblot analysis of SHP2 and Parkin in mitochondrial and cytosolic components from SH-SY5Y cells pretreated with lovastatin (10 μM) for 3 h followed by the addition of 30 μM rotenone for 3 h. (b, c) Immunofluorescence analysis of co-localization of SHP2 (red) and Tom20 (green) or Parkin (red) and Tom20 (green) from SH-SY5Y cells treated with lovastatin (10 μM) followed by the addition of 30 μM rotenone for 3 h. Scale bar represents 10 μm. Data are representative of three independent experiments. **P*<0.05 vs. as rotenone-treated group.


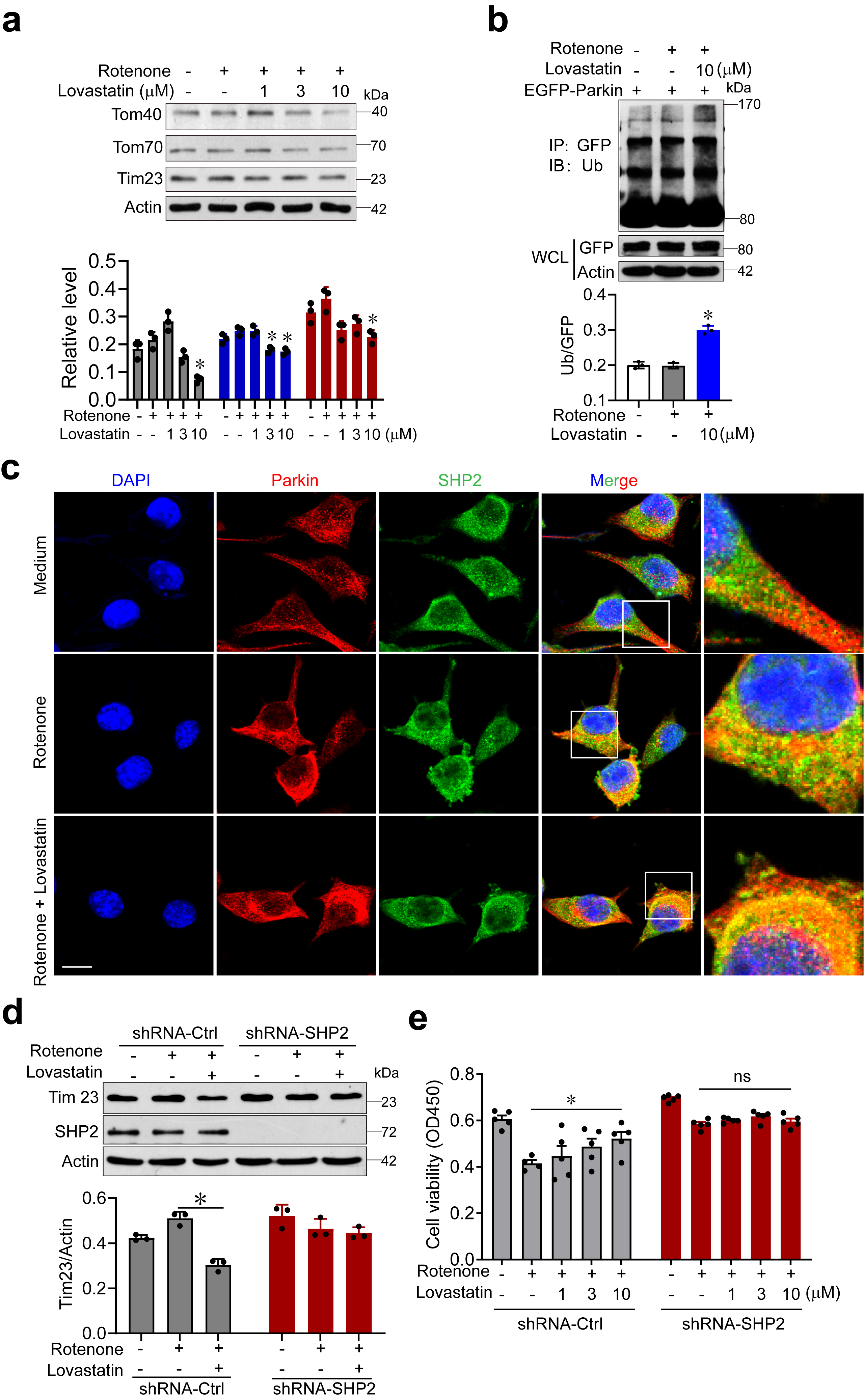


Figure. S9.

**Parkin-mediated mitophagy triggered by lovastatin depends on SHP2. (a)** Immunoblot analysis of mitochondrial protein expression in SH-SY5Y cells pretreated with 1, 3, 10 μM lovastatin for 3 h followed by the addition of 30 μM rotenone for 12 h. **(b)** Co-IP analysis of Parkin ubiquitination in SH-SY5Y cells transfected with EGFP-Parkin for 24 h followed by the addition of lovastatin and rotenone (30 μM, 6 h). **(c)** Immunofluorescence analysis of co-localization of SHP2 (green) and Parkin (red) from SH-SY5Y cells pretreated with 10 μM lovastatin for 3 h followed by the addition of 30 μM rotenone for 6 h. Scale bar represents 10 μm. **(d)** Immunoblot analysis of mitochondrial protein expression in shRNA-Ctrl or shRNA-SHP2 SH-SY5Y cells after being treated with 10 μM lovastatin for 3 h followed by the addition of 30 μM rotenone for 12 h. **(e)** Cell viabilities were measured by CCK8 assay in shRNA-Ctrl or shRNA-SHP2 SH-SY5Y cells after being treated with 1, 3, 10 μM lovastatin for 3 h followed by the addition of 30 μM rotenone for 6 h. Data are representative of three independent experiments (mean ± SEM). **P*<0.05 vs. as indicated.


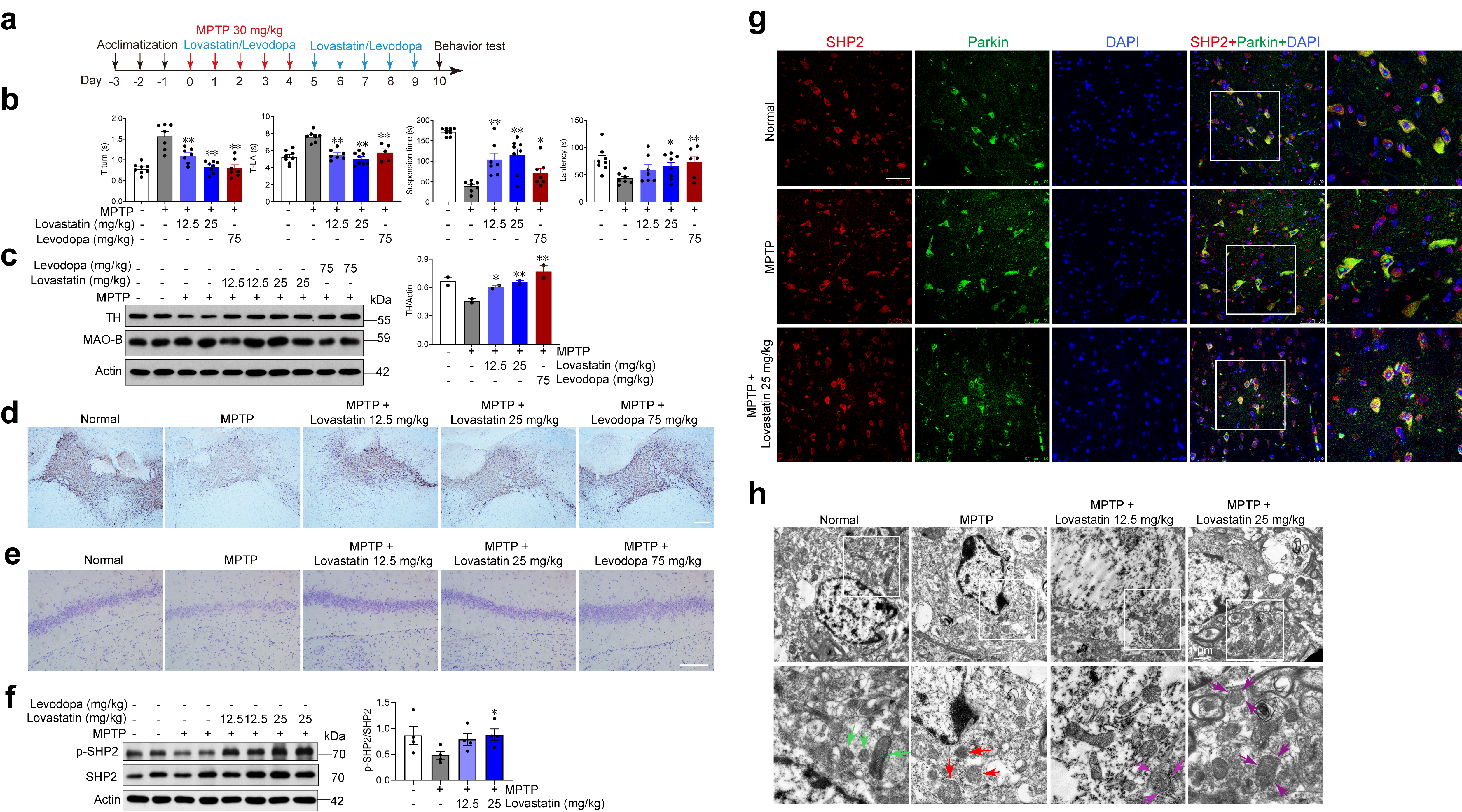


Figure. S10.

**Lovastatin ameliorates motor deficits by promoting SHP2-mediated mitophagy in MPTP-challenged mice.** (**a**) Overview of the experimental design. (**b**) The pole test was used to measure the motor function. The time from when the mice were positioned head-upward near the top of the pole until they turned completely downward was recorded and indicated as T turn, while the total time taken in the pole test was indicated as T-LA. Beam hang test was used to measure the grip strength and motor coordination (suspension time). The effect of lovastatin on the fall latency of rotarod test in the MPTP-induced PD mice model (latency time). (**c, d**) The expressions of TH in the striatum of each group were measured using Western blot analysis and immunohistochemical staining. (**e**) Neuronal loss in striatum was measured by Nissl staining. **(f)** Western blot analysis of p-SHP2 and SHP2 in the striatum of each group. (**g**) Co-localization of SHP2 and Parkin in the striatum were analyzed via immunofluorescence stain. (**h**) The mitophagy was assessed by TEM. Scale bar 50 μm in **d**, **e** and **g**. Scale bar 1 μm in **h**. Data represent mean ± SEM, n=7. **P*<0.05, ***P*<0.01 *vs.* MPTP group.


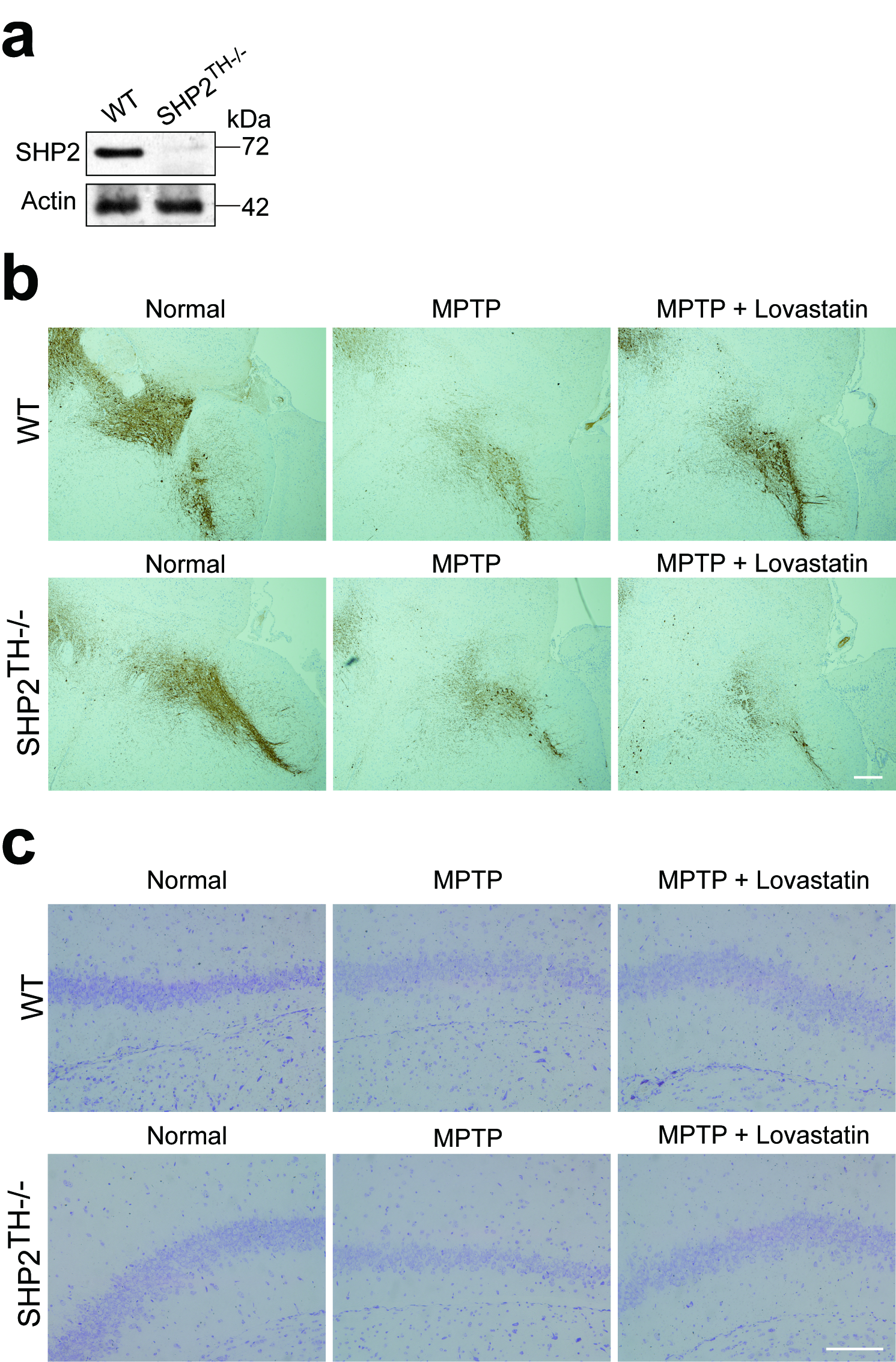


Figure. S11.

**The ameliorative effect of lovastatin in MPTP-challenged mice is significantly blocked by SHP2 deficiency in dopaminergic neurons.** (**a**) The expressions of SHP2 in the striatum of WT and SHP2^TH-/-^ mice were measured using western blot analysis. (**b**) The expressions of TH in the striatum of each group were measured using immunohistochemical staining. (**c**) Neuronal loss in striatum was measured by Nissl staining. Scale bar 50 μm in **b** and **c**.
